# Laser vs rotational transvenous lead extraction: A systematic review and meta-analysis of procedural safety and efficacy outcomes

**DOI:** 10.1016/j.hroo.2026.01.003

**Published:** 2026-01-12

**Authors:** Alphonsus C. Liew, Nadeev Wijesuriya, Felicity de Vere, Sandra Howell, Vishal Mehta, Steven Niederer, Christopher Aldo Rinaldi

**Affiliations:** 1 School of Biomedical Engineering and Imaging Sciences, King’s College London; 2 Department of Cardiology, Guy’s and St Thomas’ NHS Foundation Trust, London, United Kingdom; 3 National Heart and Lung Institute, Imperial College London, London, United Kingdom; 4 The Alan Turing Institute, London, United Kingdom

**Keywords:** Transvenous lead extraction, Laser, Rotational, Pacemaker, Implantable cardiac defibrillator, Meta-analysis

## Abstract

**Background:**

The rise in cardiac implantable electronic devices implantation has resulted in a concomitant rise in transvenous lead extractions (TLEs). Advanced extraction tools such as the excimer laser and mechanical rotational sheaths have improved acute procedural success when manual extraction fails. However, it is unclear how the laser sheath compares with the rotational sheath in safety and efficacy outcomes.

**Objective:**

We aimed to compare the safety and efficacy of laser and rotational TLEs using a meta-analysis.

**Methods:**

A systematic literature search was performed for studies involving the use of laser and/or rotational sheath published from 2008 onward. A random-effects model was used to compare outcome data including complete procedural success, clinical success, major complications, minor complications, and procedural death.

**Results:**

43 studies were included for meta-analysis, consisting of 13,189 patients and 20,103 extracted leads. The overall mean lead dwell time was 8.5 ± 12.7 years. There was no significant difference between laser- and rotational-assisted TLEs in complete procedural success (94% vs 94%, respectively; *P* = .92), major complications (1.9% vs 0.81%, respectively; *P* = .10), minor complications (5% vs 4%, respectively; *P* = .74), and procedural death (0.2% vs 0.04%, respectively; *P* = .14). Laser TLE had a 5.6-fold aggregated risk of SVC laceration compared with rotational TLE but this was not associated with an increased mortality.

**Conclusion:**

Both laser and rotational TLEs are effective and safe. Our analysis suggests that there is no significant difference in safety profile between laser and rotational TLEs.

**Key Findings**

▪ This meta-analysis incorporates contemporary transvenous lead extraction (TLE) registries such as GALLERY and PROMET.
▪ There is no statistically significant difference in procedural death, major complications, and minor complications between laser TLE and rotational TLE.
▪ Both laser and rotational TLE had similar complete procedural and clinical success rates.
▪ Laser TLE had higher aggregated superior vena cava laceration rates than rotational TLE but this was not statistically significant.
▪ Low-volume centers were associated with a higher risk of procedural death than high-volume centers.

## Introduction

Over the last decade, transvenous lead extractions (TLEs) have been increasingly performed, in line with the increasing prevalence of cardiac implantable electronic devices. Furthermore, the age and number of extracted leads are also increasing, making extraction procedures more challenging, often necessitating the use of powered sheaths such as laser and rotational mechanical sheaths. Both laser and rotational sheaths have been shown to be safe and effective in achieving complete procedural success, defined as the complete removal of all targeted leads and lead material from the vascular space with the absence of permanently disabling complication or procedure-related death.^1^ However, how laser-assisted TLE compares with rotational-assisted TLE in safety and efficacy remains unclear. 2 meta-analyses have compared laser with rotational TLE,^2^^,^^3^ but both have inherent biases such as the inclusion of studies without tool-specific outcomes and the omission of large contemporary registries.^4^, ^5^, ^6^, ^7^ Moreover, the meta-analysis by Lee et al^3^ included studies exclusively investigating high-risk cohorts such as superior vena cava obstruction, octogenarians, pediatric population, and arrhythmogenic cardiomyopathy using only laser sheaths, likely skewing outcomes for laser-assisted TLE. We aimed to perform a meta-analysis that incorporates contemporary outcome data to provide an accurate and up-to-date safety and efficacy profile of laser and rotational TLEs.

## Methods

### Literature search

This meta-analysis was registered on PROSPERO (ID: CRD420251119895). A systematic literature search was conducted according to the Preferred Reporting Items for Systematic Reviews and Meta-Analyses guidance.^8^ PubMed and Embase databases were searched for clinical studies published between January 1989 and March 2025 using Boolean operators and the search terms “extraction”, “removal”, “laser”, “excimer laser”, “laser-assisted”, “Glidelight”, “rotational”, “mechanical”, “Evolution”, “Tightrail”, “powered sheath”, “rotating sheath”, “pacemaker”, “CIED”, “defibrillator”, “lead”, “transvenous lead”, and “catheter”. Full search terms can be found in the Supplemental Material.

### Search criteria

Duplicates were screened and removed. After the initial literature search, to minimize temporal bias, we decided to exclude studies published before the introduction of mechanical rotational sheaths in 2008. Accordingly, studies that met the following criteria were excluded from meta-analysis: patient cohort <18 years of age, systematic reviews, letters, editorials, case series with <10 patients, case reports, studies published before 2008, surveys, conference abstracts, review articles, nonhuman trials, overlapping study population, unrelated to TLE, congenital heart disease cohort, studies not published or translated into English, concomitant cardiac surgery, procedural success rate, or mortality rate not reported.

### Screening

2 authors (A.C.L. and N.W.) independently performed screening of the studies produced from the literature search. Where there was a screening conflict that could not be resolved between the 2 authors, C.A.R. adjudicated on the inclusion of the conflicted study. Study records were first screened using their titles and abstracts to filter out duplicates and studies that met the exclusion criteria. Full-text articles of the remaining studies were subsequently obtained and screened in detail for inclusion into the meta-analysis. Studies lacking tool-specific outcome data, including those reporting combined laser and rotational TLEs or combined laser and manual traction–only results without stratification by an extraction tool, were excluded.

### Definitions

The Heart Rhythm Society and European Heart Rhythm Association consensus statements were used for the definition of complete procedural success, clinical success, major complication, minor complication, and procedure-related death.^1^^,^^9^ Where the definitions used in studies differed, the outcomes were reclassified based on the Heart Rhythm Society and/or European Heart Rhythm Association consensus definitions. Centers that performed ≥30 TLE procedures a year were classed as high-volume centers, and those that performed <30 procedures a year were classed as low-volume centers.

### Statistical analysis

Baseline characteristics were presented as combined mean and standard deviation, calculated using the Cochrane formula.^10^ A meta-analysis was performed based on the extraction tool (laser or rotational) to compare the proportion of complete procedural success, major complications, minor complications, and procedural death between the groups. A random-effects model with the restricted maximum likelihood method was used to pool the proportion for each group to account for interstudy heterogeneity. The Freeman-Tukey double arcsine transformation was applied before pooling to stabilize variances and reduce the impact of extreme proportions. Heterogeneity was assessed using Cochrane’s Q test and quantified using the I^2^ statistic. Leave-one-out analyses were performed to assess the impact of individual studies on the pooled estimate. To explore sources of heterogeneity, meta-regressions were performed using baseline characteristics including age, lead dwell times, defibrillator lead, atrial fibrillation, hypertension, diabetes, previous sternotomy, infective indication, noninfective indication, and number of redundant leads. Funnel plots were generated to look for the presence of publication bias. Egger’s and Begg’s tests were used to quantify asymmetry in publications. Where publication bias was identified, a “trim-and-fill” analysis and subgroup analysis were performed to adjust for any potential publication bias.

## Results

### Literature search

**Figure 1:**
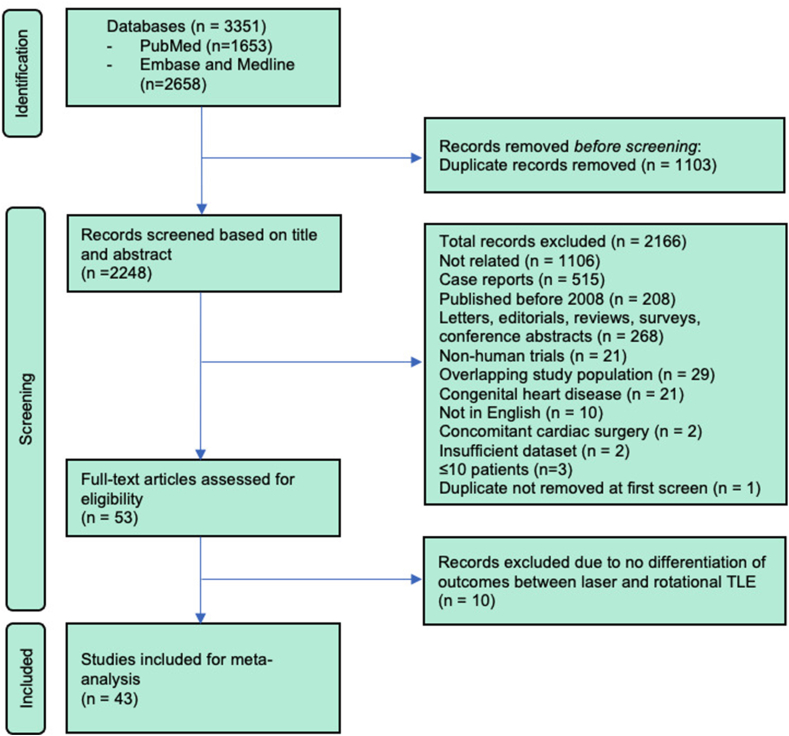
Screening flow chart for studies to be included in the meta-analysis, as per Preferred Reporting Items for Systematic Reviews and Meta-Analyses guidelines. TLE = transvenous lead extraction.

A total of 3351 records were identified from PubMed, MEDLINE, and Embase. Of these, 1103 were identified as duplicates and removed prior to screening. The remaining 2248 records were screened by reading the title and abstract. Of these, 2166 were removed in accordance with the inclusion/exclusion criteria. The remaining 53 articles underwent detailed full-text screening, culminating in the exclusion of 10 articles owing to the inability to extract tool-specific (laser or rotational) outcomes, leaving a total of 43 studies to be included in the meta-analysis (Supplemental Tables 1 and 2). 4 studies consisted of both laser- and rotational-assisted TLE cohorts and were treated as separate studies for analysis. 1 study combined 2 analyses, one of a single-center and another of a multicenter analysis. These were treated as 2 separate studies for the meta-analysis. The screening processes for the meta-analysis are presented in Figure 1.

### Baseline characteristics

Baseline characteristics are presented in Table 1. A total of 13,189 patients were included with a mean age of 66.4 ± 17.9 years and 34.1% females. Laser-assisted TLE was performed in 6992 patients and rotational-assisted TLE in 6197 patients. Overall, 19,530 leads were extracted with a mean lead dwell time of 8.5 ± 8.6 years. Infective indication was the most common indication (59.3%), followed by lead dysfunction (27.7%). The most frequently extracted lead type was pacemaker leads (52%), followed by defibrillator leads (30.9%) and left ventricular leads (2.9%).

**Table 1:** Aggregated baseline characteristics

	Total (13,189)	Laser (n = 6992)	Rotational (n = 6197)	Laser pooled proportion	Rotational pooled proportion	*P* value∗
**Number of patients**	13,189	6992 (53%)	6197 (47%)			
**Mean age (y)**	66.4 ± 17.9	66.4 ± 16.5	64.6 ± 19.4			
**Female**	4504 (34.1%)	1835 (26.2%)	2669 (43.1%)			
**Extracted leads**	19,530	12,026	7504			
**Indication**						
Infective	7792/13,150 (59.3%)	4525/6953 (65.1%)	3267/6197 (52.7%)	0.70 (0.63–0.77)	0.64 (0.5–0.68)	.06
Lead dysfunction	2939/10,597 (27.7%)	1503/6671 (22.7%)	1436/3926 (36.6%)			
Device upgrade	230/10,074 (2.3%)	121/6409 (1.9%)	109/3665 (3.0%)			
Vein occlusion	102/8806 (1.2%)	91/5874 (1.5%)	11/2932 (0.4%)			
Redundant leads	406/9361 (4.3%)	405/6096 (6.6%)	1/3265 (0.03%)			
Significant TR	25/9139 (0.3%)	22/5874 (0.4%)	3/3265 (0.1%)			
Lead dwell time (y)	8.5 ± 8.6	8.6 ± 13.8	8.2 ± 5.6			
**Lead type**						
PPM	3150/6058 (52.0%)	1613/3686 (43.8%)	1537/2372 (64.8%)	0.31 (0.19–0.44)	0.31 (0.21–0.43)	.95
ICD	1870/6058 (30.9%)	1255/3686 (34.0%)	615/2372 (25.9%)	0.40 (0.2–0.62)	0.24 (0.19–0.29)	.13
LV lead	177/6058 (2.9%)	139/3686 (3.8%)	38/2372 (1.6%)	0.03 (0.01–0.06)	0.05 (0.04–0.09)	.13

ICD = implantable cardiac defibrillator; LV = left ventricular; PPM = pacemaker; TR = tricuspid regurgitation.

∗ Comparison between pooled laser and rotational groups.

### Efficacy outcomes

**Figure 2:**
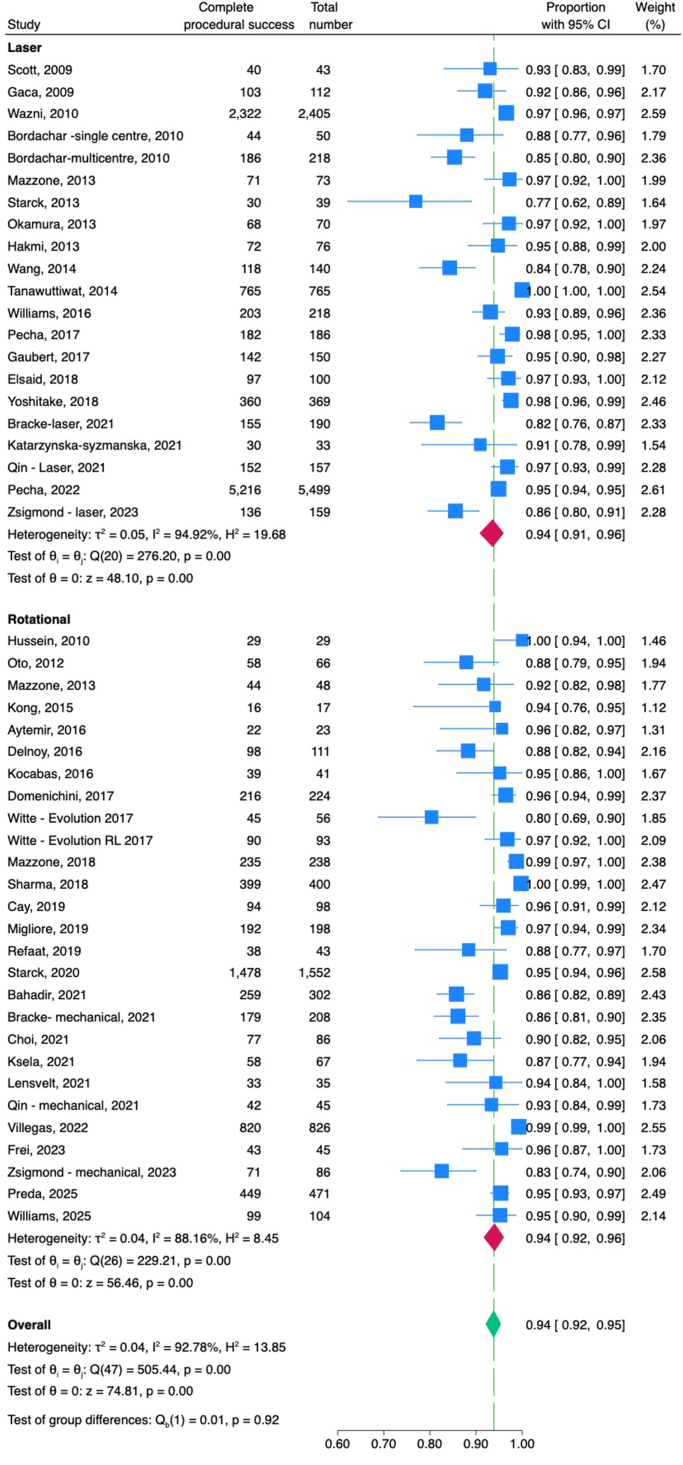
Forest plot of complete procedural success rates with laser and rotational transvenous lead extraction. CI = confidence interval.

Complete procedural success was reported either per patient or per lead, depending on the study. Overall, complete procedural success was 94% (confidence interval 92%–95%), and the clinical success rate was 98% (confidence interval 96%–99%). Laser TLE was associated with a similar complete procedural success rate as rotational TLE (94% vs 94%; *P* = .92) (Figure 2). Similarly, there was no difference in clinical success between laser and rotational groups (98% vs 98%; *P* = .93) (Supplemental Figure 1).

### Safety outcomes

Safety outcomes are presented in Table 2. Owing to the small number of studies reporting a breakdown of major and minor complications, we did not perform a meta-analysis of pooled proportions for each specific complication. Major complications including death occurred in 242 cases in total with an aggregated rate of 1.8%. SVC laceration occurred in 87 cases with an aggregated rate of 0.7%. Minor complications occurred in 334 cases with an aggregated rate of 2.5%. Procedural deaths occurred in 52 cases with an aggregated rate of 0.4%.

**Table 2:** Aggregated and pooled major and minor complications

Complication	Count (n = 13,189)	Laser aggregated (n = 6992)	Rotational aggregated (n = 6197)	Laser pooled proportion	Rotational pooled proportion	*P* value∗
**Major complications**	242 (1.83%)	144 (2.06%)	74 (1.19%)	1.90% (1.2–2.8)	0.8% (0.28–1.50)	.10
Procedural death	52 (0.39%)	39 (0.56%)	13 (0.21%)	0.20% (0.04–0.40)	0% (0–0.06)	.14
SVC laceration	85/10,897 (0.78%)	75/6992 (1.07%)	17/3905 (0.44%)	0.41% (0.13–0.80)	0.01% (0.00–0.23)	.14
Studies ≤ 2016	20/3632 (0.55%)	18/3228 (0.56%)	2/404 (0.50%)	0.80% (0.22–1.6)	0.03% (0–0.33)	.10
Studies > 2016	67/7265 (0.92%)	57/3764 (1.51%)	10/3501 (0.29%)	0.2% (0.01–0.54)	0.04% (0–0.89)	.82
SVC-related deaths†	21/7890 (0.27%)	18/2713 (0.66%)	2/2438 (0.08%)	0.05% (0–0.24)	0% (0–0)	.14
Studies ≤ 2016	5/2827 (0.18%)	5/2713 (0.18%)	0/114 (0%)	0.34% (0.03–0.88)	0% (0–0)	.11
Studies > 2016	16/5063 (0.32%)	14/2739 (0.51%)	2/2324 (0.09%)	0.001% (0–0.12)	0% (0–1.7)	.74
Cardiac tamponade	39 (0.30%)	22 (0.31%)	17 (0.27%)			
Hemothorax	8 (0.06%)	6 (0.09%)	2 (0.03%)			
Cardiac avulsion	18 (0.14%)	15 (0.21%)	3 (0.05%)			
Right atrial perforation	8 (0.06%)	3 (0.04%)	5 (0.08%)			
Right ventricular perforation	5 (0.04%)	1 (1.43%)	4 (0.06%)			
Subclavian vein injury requiring emergency repair	2 (0.02%)	2 (0.03%)	0			
Severe tricuspid regurgitation	9 (0.07%)	2 (0.03%)	7 (0.11%)			
Stroke	6 (0.05%)	1 (0.01%)	5 (0.08%)			
IVC laceration	1 (0.01%)	1 (0.01%)	0			
Life-threatening hemorrhage	1 (0.01%)	0	1 (0.02%)			
Other	6 (0.05%)	1 (0.01%)	5 (0.08%)			
**Minor complications**	334 (2.53%)	204 (2.92%)	130 (2.10%)	5% (2–9)	4% (3–6)	.74
Pericardial effusion not requiring drainage	34 (0.26%)	15 (0.21%)	19 (0.31%)			
Worsened TV function (not severe)	8 (0.06%)	2 (0.03%)	6 (0.10%)			
Hematoma	178 (0.01%)	110 (1.26%)	68 (1.10%)			
Pneumothorax	23 (0.17%)	12	11 (0.18%)			
Hemorrhage	22 (0.17%)	12	10 (0.16%)			
Pocket abscess	1 (0.76%)	1	0			
Pulmonary embolism not requiring thrombolysis	8 (0.06%)	6	2 (0.03%)			
Hepatic vein embolism not requiring intervention	0	0	0			
Subclavian vein thrombosis	13 (0.10%)	10	3 (0.05%)			
Deep vein thrombosis	9 (0.07%)	4	5 (0.08%)			
Pleural effusion	24 (0.18%)	23	1 (0.02%)			
Arrhythmia	9 (0.07%)	9	0			
Vascular repair at the femoral access site	4 (0.03%)	0	4 (0.06%)			
Pulmonary contusion	1 (0.01%)	0	1 (0.02%)			

IVC = inferior vena cava; SVC = superior vena cava; TV = tricuspid valv*e*.

∗ P value comparing pooled laser and rotational outcomes.

† Studies that had SVC laceration events.

**Figure 3:**
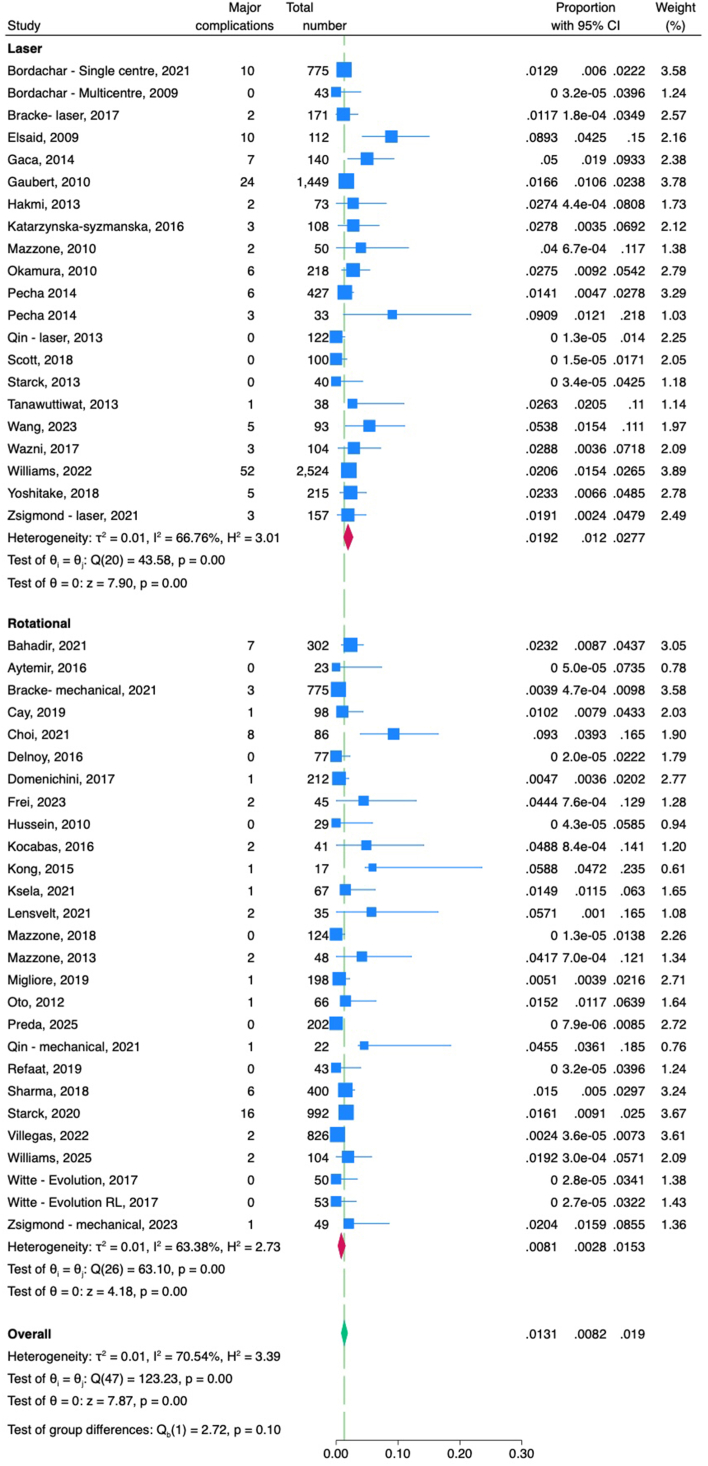
Forest plot comparing major complications between laser and rotational transvenous lead extraction. CI = confidence interval.

**Figure 4:**
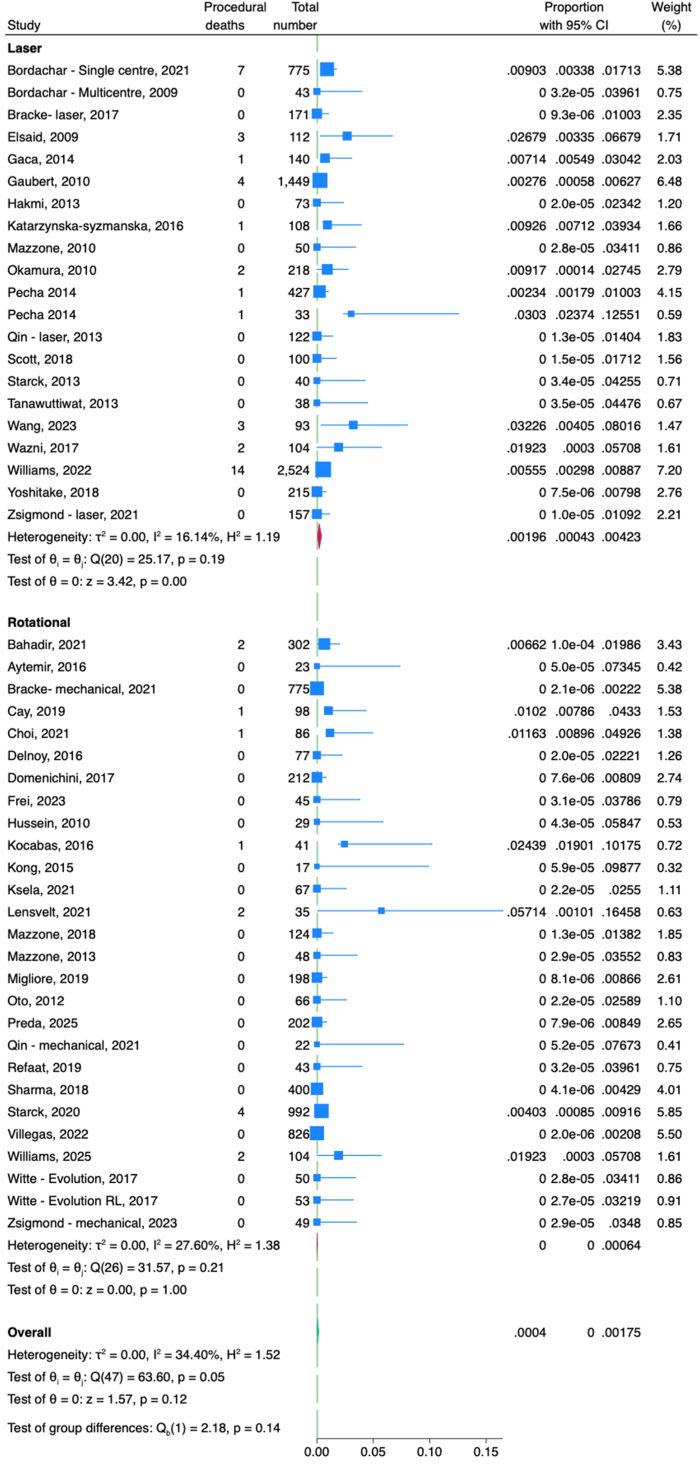
Forest plot comparing procedural death rates between laser and rotational transvenous lead extraction. CI = confidence interval.

The pooled rate of major complications including death did not differ significantly between laser- and rotational-assisted TLE groups (1.9% vs 0.8%, respectively; *P* = .10) (Figure 3). Similarly, there was no significant difference in the pooled rate of minor complications between laser and rotational (5% vs 4%, respectively; *P* = .74) (Supplemental Figure 2). The pooled rate of procedural deaths also did not differ significantly between the laser and rotational groups (0.2% vs 0%, respectively; *P* = .14) (Figure 4).

Laser-assisted TLE demonstrated a 5.6-fold higher aggregated risk of SVC laceration than rotational TLE. However, this difference did not reach statistical significance when pooled proportions were compared (*P* = .14) (Supplemental Figure 3). To further explore temporal trends, studies reporting SVC laceration were stratified into studies with cases performed up to 2016—prior to the introduction of the vascular occlusion balloon—and those including cases performed after 2016. Although laser-assisted TLE showed a trend toward higher pooled proportions of SVC laceration than rotational TLE in both the pre- and post-2016 periods, these differences were not statistically significant (Supplemental Figures 4 and 5). In addition, SVC-related death rates similarly did not differ significantly between groups in either time period (Supplemental Figure 6).

### Sensitivity analyses

Leave-one-out analyses produced no significant skew in pooled complete procedural success, clinical success, major complications, minor complications, or procedural death. Meta-regression analyses did not identify statistically significant associations between baseline characteristics (lead dwell time, infective indication, implantable cardiac defibrillator [ICD] lead, age, diabetic status, chronic kidney disease, hypertension, atrial fibrillation, coronary artery disease, and previous sternotomy) and safety and efficacy outcomes.

### Publication bias

#### Major complications

**Figure 5:**
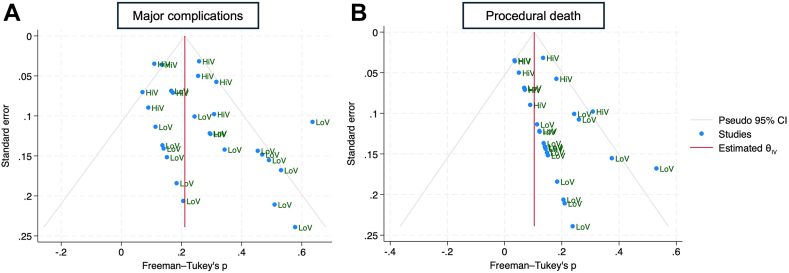
Funnel plot showing asymmetry in publications (blue dots) in the reporting of **(A)** major complications and **(B)** procedural deaths in the rotational subgroup. In both funnel plots, there is a preponderance of low-volume centers reporting higher complication and procedural death rates, respectively. The right side of the red line denotes higher rates of outcomes, and the left side of the red line denotes lower rates of outcomes. CI = confidence interval; HiV = high-volume center; LoV = low-volume center.

The funnel plot for major complications revealed asymmetry in publications with a greater number of small publications reporting higher major complication rates in the rotational TLE subgroup (Figure 5A). Similarly, Egger’s and Begg’s tests revealed statistically significant small-study effects (Egger’s *P* = .01; Begg’s *P* = .02) in the rotational subgroup. To explore this asymmetry, we conducted a subgroup analysis of high- vs low-volume centers to determine whether these effects were attributable to center volume rather than publication bias (Supplemental Figure 7). This analysis revealed that low-volume centers had significantly higher major complication rates than high-volume centers (1.9% vs 1.1%, respectively; *P* = .03), suggesting that the funnel plot asymmetry with low-volume centers reporting a high major complication rate reflects a true effect rather than bias in the literature.

#### Procedural death

The funnel plot for procedural death in the rotational TLE subgroup suggested the presence of publication bias, with a preponderance of smaller studies reporting higher procedural deaths (Figure 5B). Egger’s and Begg’s tests confirmed this small-study effect on the overall outcome. Again, to explore this asymmetry, we conducted a subgroup analysis of procedural deaths between high- and low-volume centers (Supplemental Figure 8). This demonstrated higher procedural death rates in low-volume centers than high-volume centers (0.2% vs 0.1%, respectively; *P* = .01). This suggests that the funnel plot asymmetry likely reflects a true effect of smaller studies, which predominantly consisted of low-volume centers, on procedural death (ie, low-volume centers associated with a higher risk of procedural death).

## Discussion

Our meta-analysis differs from the meta-analyses performed previously by Akhtar et al^2^ and Lee et al^3^ in several aspects. First, our meta-analysis included large contemporary registries currently available that were not included in previous meta-analyses, such as the German TLE registry (GALLERY) and the Patient-Related Outcomes of Mechanical Lead Extraction Techniques registry.^4^^,^^5^ Our meta-analysis also included significantly more rotational TLE studies (n = 27) than the meta-analyses by Lee et al^3^ (n = 14) and Akhtar et al^2^ (n = 12), improving the robustness of our findings. Second, although previous meta-analyses included studies where tool-specific outcomes could not be separated from the overall outcomes,^11^, ^12^, ^13^, ^14^ we only included studies where tool-specific outcomes could be determined, thus ensuring that reported outcomes can be completely attributed to either laser or rotational sheath use, Third, we included only studies published after 2008, when mechanical rotational sheaths were introduced. This was to minimize temporal bias from studies published as early as 1994 that may be caused by undetected differences in demographics, medical therapy, technology, or changes in procedural workflow, providing a fairer comparison between laser and rotational TLEs. Fourth, in contrast to the meta-analysis by Lee et al,^3^ we have also excluded studies that investigated TLE in high-risk groups—such as the geriatric, pediatric, SVC obstruction, and arrhythmogenic cardiomyopathy populations—in only 1 arm of the cohort studied, because this is likely to introduce selection bias, which could skew outcomes.^15^, ^16^, ^17^, ^18^

The results of our meta-analysis suggest that laser and rotational TLEs are both safe and effective. Both laser and rotational TLEs have similar rates of complete procedural success, clinical success, major complications, procedural deaths, and minor complications. Notably, the complete procedural (94%) and clinical success rates (98%) in our meta-analysis were higher than those reported in previous meta-analyses.^2^^,^^3^ This is likely caused by the inclusion of the more recent large GALLERY, involving 2524 patients using laser extraction, which reported a similar complete procedural success rate of 94.9% and clinical success rate of 97.9%.^4^ Although we did not include the large multicenter European TLE registry in our meta-analysis owing to the absence of tool-specific outcomes being reported, the aggregated rates of major complications (1.8%) and procedural death (0.4%) in our study were very similar to the European TLE registry (1.9% and 0.5% respectively), supporting the robustness of our meta-analysis.^19^

Evidence of publication bias and funnel plot asymmetry was observed in the rotational subgroup, largely owing to a concentration of small studies reporting a higher rate of major complications and procedural mortality rates. To explore this asymmetry, we conducted subgroup analyses comparing high- and low-volume centers. These analyses demonstrated that low-volume centers had significantly higher rates of major complications and procedural deaths than high-volume centers. This suggests that detected asymmetry reflects true effects related to center volume—particularly among smaller studies, which were predominantly low-volume centers—rather than publication bias.

Our overall analysis suggests that there is no significant difference in safety profile between laser and rotational TLEs. This is consistent with the findings of Akhtar et al’s^2^ meta-analysis, which found no significant difference in major complications or procedural death between laser and rotational TLEs.

Our findings contrast with the meta-analysis by Lee et al^3^ that reported a 9.3-fold higher risk of death with laser sheaths than rotational sheaths. This discrepancy may be explained by several factors. The time frame used in our study differed from Lee et al’s^3^ meta-analysis, which included studies published between 1998 and 2007 for laser TLE and studies published between 2009 and 2017 for rotational TLE. This may have introduced a temporal bias where earlier laser TLE studies reported higher mortality rates owing to less operator experience and less refined TLE workflow. The current study included the results of large contemporary TLE registries such as GALLERY (laser) and Patient-Related Outcomes of Mechanical Lead Extraction Techniques (rotational subgroup), which have demonstrated improved safety outcomes in both groups.^4^^,^^5^

There was a trend toward a higher proportion of ICD leads in the laser group than the rotational group, although this was not statistically significant (pooled proportions 0.4 vs 0.24; *P* = .13). A higher incidence of SVC injury would be expected with ICD leads.

Finally, we found a greater trend in SVC laceration with the laser TLE group but this was not statistically significant. This contrasts with the findings of previous meta-analyses by Akhtar et al^2^ and Lee et al,^3^ which found a statistically significant difference in SVC laceration rates. This difference is likely explained by the greater proportion of rotational TLE-related SVC laceration rates in the newer studies not included in previous meta-analyses. For example, the aggregated rate of SVC laceration with rotational TLE was 0.44% in our meta-analysis vs 0.18% in Akhtar et al’s^2^ analysis, whereas the rates for laser TLE were the same in both meta-analyses (1.07%).

The introduction of the SVC occlusion balloon in 2016—which has been shown to significantly reduce mortality associated with SVC laceration^20^—would be expected to have led to improved outcomes in the laser TLE group in the post-2016 era. However, our subgroup meta-analysis comparing studies conducted before and after 2016 demonstrated no statistically significant differences in procedural mortality or SVC-related deaths. Although the SVC occlusion balloon has been available since 2016, its adoption may not have been universal across centers. Moreover, insufficient reporting precluded stratification of studies according to actual balloon use, which may have influenced the pooled results. The lack of statistical significance for SVC-related mortality may also reflect the exceptionally low incidence of these events overall, limiting the statistical power to detect meaningful differences.

### Limitations

Owing to the scarcity of randomized controlled trials comparing laser and rotational TLEs, our meta-analysis comprised mostly observational studies reporting outcomes associated with 1 type of powered sheath, either laser or rotational, introducing the possibility of selection bias. Attempts to minimize this have been taken, including the exclusion of 1-armed studies investigating the use of laser or rotational sheaths in high-risk groups such as the pediatric or geriatric population, superior vena cava obstruction, and arrhythmogenic cardiomyopathy.

A significant number of studies did not report on tool-specific outcomes. Our meta-analysis is the first to exclude all such studies to remove the confounding effect of nonspecific outcomes on our overall results.

There was a high degree of interstudy heterogeneity in our meta-analyses of complete procedural success and clinical success. To account for this, we performed meta-regressions, which did not identify an effect of lead dwell time or baseline characteristics on the overall outcomes.

Baseline characteristics data such as comorbidities and TLE indication were not reported in all the studies analyzed. Therefore, we were only able to conduct meta-regression in the studies with complete baseline characteristics data, which may have affected the results of our meta-regression.

We detected significant publication bias in the rotational TLE subgroup, arising from a disproportionate number of small studies reporting a higher incidence of major complications and procedural death. We were able to explain this by performing a subgroup analysis of high- vs low-volume centers, demonstrating that the asymmetry observed likely represented the true effect of centers with low volume on safety outcomes.

Crossover from 1 powered sheath to another may have affected outcomes in our analysis. However, only 2 studies reported crossover from 1 powered sheath to another, and the effect of this on the overall outcome is likely to be minimal.

## Conclusion

Our meta-analysis provides robust and contemporary evidence that laser and rotational TLEs have similarly high rates of safety and efficacy.

## Disclosures

The N.W., F.d.V., A.C.L., and S.H. are supported by the Wellcome/Engineering and Physical Sciences Research Council Center for Medical Engineering (WT203148/Z/16/Z). N.W. has received funding from the British Heart Foundation (FS/CRTF/22/24362) and travel funding from EBR Systems. S.N. acknowledges support from the UK Engineering and Physical Sciences Research Council (EP/M012492/1,NS/A000049/1,EP/P01268X/1), the British Heart Foundation (PG/15/91/31812,PG/13/37/30280,SP/18/6/33805), US National Institutes of Health (NIH R01-HL152256), and European Research Council (ERC PREDICT-HF 864055). C.A.R. receives research funding and/or consultation fees from Abbott, Medtronic, Boston Scientific, Spectranetics, EBR Systems, and MicroPort.

## Footnotes

Appendix Supplementary data associated with this article can be found in the online version at https://doi.org/10.1016/j.hroo.2026.01.003.
