# Supplementary material for: Laser vs rotational transvenous lead extraction: A systematic review and meta-analysis of procedural safety and efficacy outcomes
Source: Heart Rhythm O2. 2026 Jan 12;7(3):494–504. doi: 10.1016/j.hroo.2026.01.003 (PMC13031037; doi:10.1016/j.hroo.2026.01.003)
Supplement: Supplementary File [file mmc1.docx]

**1. Search terms:**

(specified to be 1989-current year):

1. Pubmed search re: rotational extraction

*(CIED OR pacemaker OR defibrillator OR catheter OR lead or transvenous lead) AND (removal OR extraction) AND (mechanical sheath OR rotational sheath OR powered sheath OR rotating sheath OR dilator sheath OR Evolution RL OR Evolution sheath OR TightRail)*

1. Pubmed search re: laser extraction

*(CIED OR electrode OR pacemaker OR defibrillator OR catheter OR lead) AND (removal OR extraction) AND* *(Laser-assisted OR excimer laser OR laser sheath OR GlideLight or Laser adj2 extraction)*

1. Embase search re: rotational extraction

*((defibrillator/ or cardiac rhythm management device/ or cardiovascular therapeutic device/ or life support equipment/ or defibrillator accessory/ or defibrillator pacemaker/ or high energy defibrillator/ or implantable cardioverter defibrillator/ or internal defibrillator/ or low energy defibrillator/) OR (defibrillat*) OR (cardiovascular therapeutic device*) OR (catheter removal/ or catheter sheath/) OR ((catheter or lead) OR (pacemaker*) OR (electrode*) OR (CIED)))*

*AND*

*(remov* or extract*)*

*AND*

*(((mechanical adj2 sheath*).mp) OR ((rotat* adj2 sheath*).mp) OR ((power* adj2 sheath*).mp) OR ((dilat* sheath*).mp) OR ((Evolution adj2 RL).mp) OR ((****Evolution adj2 sheath****).mp) OR (TightRail.mp))*

1. Embase search re: laser extraction

*((defibrillator/ or cardiac rhythm management device/ or cardiovascular therapeutic device/ or life support equipment/ or defibrillator accessory/ or defibrillator pacemaker/ or high energy defibrillator/ or implantable cardioverter defibrillator/ or internal defibrillator/ or low energy defibrillator/) OR (defibrillat*) OR (cardiovascular therapeutic device*) OR (catheter removal/ or catheter sheath/) OR ((catheter or lead) OR (pacemaker*) OR (electrode*) OR (CIED)))*

*AND*

*(remov* or extract*)*

*AND*

*((Laser-assisted) OR (excimer laser/) OR (laser adj2 sheath) OR (GlideLight))*

| **Author, year of publication** | **High- or low-volume centre** | **DOI** | **Patient number** | **leads extracted** | **Mean pt age (years)** | **Female (%)** | **Complete procedural success (%)** | **Clinical success (%)** | **Major complications (%)** | **Procedural death (%)** |
| --- | --- | --- | --- | --- | --- | --- | --- | --- | --- | --- |
| Bracke-laser, 2021 | High | [10.1007/s12471-021-01652-w](https://doi.org/10.1007/s12471-021-01652-w) | 775 | 190 | 70.3 | 195 (25.2) | 150/190 (81.6) | 175/184 (95.1) | 10 (1.3) | 7 (0.9) |
| Scott, 2009 | Low | <https://doi.org/10.1093/europace/eup263> | 43 | 80 | 66 | 14 (32.6) | 40/43 (93.0) | Na | 0 (0.0) | 0 (0.0) |
| Pecha, 2017 | Low | <https://doi.org/10.1093/icvts/ivw298> | 171 | 186 | 58.2 | 50 (29.2) | 182/186 (97.8) | 169/171 (98.8) | 2 (1.2) | 0 (0.0) |
| Gaca, 2009 | Low | [10.1016/j.athoracsur.2009.02.015](https://doi.org/10.1016/j.athoracsur.2009.02.015) | 112 | 205 | 59.8 | 31 (27.7) | 103/112 (92.0) | Na | 10 (8.9) | 3 (2.7) |
| Wang, 2014 | Low | <https://doi.org/10.1111/jocs.12294> | 140 | 279 | 62.6 | 30 (21.4) | 118/140 (84.3) | Na | 7 (5.0) | 1 (0.7) |
| Wazni, 2010 | High | <https://doi.org/10.1016/j.jacc.2009.08.070> | 1449 | 2405 | 63.4 | 408 (28.2) | 2322/2405 (96.5) | 1416/1449 (97.7) | 24 (1.7) | 4 (0.3) |
| Mazzone, 2013 | Low | <https://doi.org/10.1093/europace/eut126> | 73 | 127 | 60.2 | 15 (20.5) | 71/73 (97.3) | 72/73 (98.6) | 2 (2.7) | 0 (0.0) |
| Williams, 2016 | Low | [10.1503/cjs.011115](https://doi.org/10.1503/cjs.011115) | 108 | 218 | 67.2 | 29 (26.9) | 203/218 (93.1) | 105/108 (97.2) | 3 (2.8) | 1 (0.9) |
| Bordachar -single centre, 2010 | High | <https://doi.org/10.1161/CIRCEP.109.933051> | 50 | 115 | 69 | 12 (24.0) | 44/50 (88.0) | Na | 2 (4.0) | 0 (0.0) |
| Bordachar-multicentre, 2010 | High | <https://doi.org/10.1161/CIRCEP.109.933051> | 218 | 458 | 71 | 50 (22.9) | 186/218 (85.0) | Na | 6 (2.8) | 2 (0.9) |
| Tanawuttiwat, 2014 | High | <https://doi.org/10.1111/pace.12406> | 427 | 765 | 67.9 | 117 (27.4) | 765/765 (100.0) | 765/765 (100.0) | 6 (1.4) | 1 (0.2) |
| Katarzynska-syzmanska, 2021 | Low | [10.33963/KP.15983](https://doi.org/10.33963/kp.15983) | 33 | 49 | 65 | 9 (27.3) | 30/33 (90.9) | 32/33 (97.0) | 3 (9.1) | 1 (3.0) |
| Starck, 2013 | High | [10.1093/europace/eut086](https://doi.org/10.1093/europace/eut086) | 122 | 39 | 60.4 | 35 (28.7) | 30/39 (76.9) | 30/39 (76.9) | 0 (0.0) | 0 (0.0) |
| Elsaid, 2018 | Low | [10.1007/s10840-018-0422-3](https://doi.org/10.1007/s10840-018-0422-3) | 100 | 158 | 60.2 | 13 (13.0) | 97/100 (97.0) | 99/100 (99.0) | 0 (0.0) | 0 (0.0) |
| Okamura, 2013 | Low | [10.1016/j.jjcc.2013.03.012](https://doi.org/10.1016/j.jjcc.2013.03.012) | 40 | 70 | 65.5 | 14 (35.0) | 68/70 (97.1) | 70/70 (100.0) | 0 (0.0) | 0 (0.0) |
| Hakmi, 2013 | High | <https://doi.org/10.1093/icvts/ivt428> | 38 | 76 | 62 | 10 (26.3) | 72/76 (94.7) | 36/38 (94.7) | 1 (2.6) | 0 (0.0) |
| Zsigmond - laser, 2023 | Low | <https://doi.org/10.1093/europace/euac200> | 93 | 159 | 67 | 23 (24.7) | 136/159 (85.5) | 85/93 (91.4) | 5 (5.4) | 3 (3.2) |
| Gaubert, 2017 | High | <https://doi.org/10.1093/europace/euw254> | 104 | 237 | 69.2 | 35 (33.7) | 142/150 (94.7) | 58/60 (96.7) | 3 (2.9) | 2 (1.9) |
| Pecha, 2022 | High | <https://doi.org/10.1093/europace/euac056> | 2524 | 6117 | 68.07 | 631(25.0) | 5216/5499 (94.9) | 2470/2524 (97.9) | 52 (2.1) | 14 (0.6) |
| Yoshitake, 2018 | High | <https://doi.org/10.1253/circj.CJ-18-0869> | 215 | 369 | 67.1 | 63 (29.3) | 360/369 (97.6) | Na | 5 (2.3) | 0 (0.0) |
| Qin - Laser, 2021 | High | <https://doi.org/10.1111/pace.14206> | 157 | 297 | 65.1 | 51 (32.5) | 152/157 (96.8) | 154/157 (98.1) | 3 (1.9) | 0 (0.0) |

**Supplemental Table 1** List of laser TLE studies

| **Author, year of publication** | **High- or low-volume centre** | **DOI** | **pt number** | **leads extracted** | **Mean pt age** | **Female (%)** | **Complete procedural success (%)** | **Clinical success (%)** | **Major complications (%)** | **Procedural death (%)** |
| --- | --- | --- | --- | --- | --- | --- | --- | --- | --- | --- |
| Bahadir, 2021 | High | <https://doi.org/10.1111/jce.15006> | 302 | 566 | 56 | 84 (27.8) | 259/302 (85.8) | 270/302 (89.4) | 7 (2.3) | 2 (0.7) |
| Aytemir, 2016 | Low | [10.1093/europace/euv245](https://doi.org/10.1093/europace/euv245) | 23 | 42 | 59.1 | 4 (17.4) | 22/23 (95.7) | 23/23 (100.0) | 0 (0.0) | 0 (0.0) |
| Bracke- mechanical, 2021 | High | [10.1007/s12471-021-01652-w](https://doi.org/10.1007/s12471-021-01652-w) | 775 | 209 | 70.3 | 195 (25.2) | 179/208 (86.1) | 183/208 (88.0) | 3 (0.4) | 0 (0.0) |
| Cay, 2019 | Low | <https://doi.org/10.1111/pace.13755> | 98 | 163 | 63.7 | 27 (27.6) | 94/98 (95.9) | 96/98 (98.0) | 1 (1.0) | 1 (1.0) |
| Choi, 2021 | Low | <https://www.nature.com/articles/s41598-021-99901-w> | 86 | 131 | 66.3 | 39 (45.3) | 77/86 (89.5) | 80/86 (93.0) | 8 (9.3) | 1 (1.2) |
| Delnoy, 2016 | Low | [10.1093/europace/euv243](https://doi.org/10.1093/europace/euv243) | 77 | 111 | 58 | 22 (28.6) | 98/111 (88.3) | 109/111 (98.2) | 0 (0.0) | 0 (0.0) |
| Domenichini, 2017 | Low | <https://doi.org/10.1093/europace/euw162> | 212 | 389 | 65.9 | 68 (32.1) | 216/224 (96.4) | na | 1 (0.5) | 0 (0.0) |
| Frei, 2023 | Low | <https://doi.org/10.1111/jce.15970> | 45 | 87 | 64.5 | 11 (24.4) | 43/45 (95.6) | 44/45 (97.8) | 2 (4.4) | 0 (0.0) |
| Hussein, 2010 | Low | [10.1016/j.hrthm.2010.03.019](https://doi.org/10.1016/j.hrthm.2010.03.019) | 29 | 41 | 64.4 | 6 (20.7) | 29/29 (100.0) | na | 0 (0.0) | 0 (0.0) |
| Kocabas, 2016 | Low | <https://springerplus.springeropen.com/articles/10.1186/s40064-016-1987-x> | 41 | 67 | 61.5 | 11 (26.8) | 39/41 (95.1) | 40/41 (97.6) | 2 (4.9) | 1 (2.4) |
| Kong, 2015 | Low | <https://pmc.ncbi.nlm.nih.gov/articles/PMC4724015/> | 17 | 31 | 67 | 7 (41.2) | 16/17 (94.1) | 16/17 (94.1) | 1 (5.9) | 0 (0.0) |
| Ksela, 2021 | Low | [10.1093/icvts/ivaa286](https://doi.org/10.1093/icvts/ivaa286) | 67 | 131 | 61.9 | 17 (25.4) | 58/67 (86.6) | 65/67 (97.0) | 1 (1.5) | 0 (0.0) |
| Lensvelt, 2021 | Low | <https://doi.org/10.1111/pace.14094> | 35 | 173 | 71.7 | 7 (20.0) | 33/35 (94.3) | 34/35 (97.1) | 2 (5.7) | 2 (5.7) |
| Mazzone, 2018 | High | <https://doi.org/10.1093/europace/eux020> | 124 | 238 | 65 | 19 (15.3) | 235/238 (98.7) | 238/238 (100.0) | 0 (0.0) | 0 (0.0) |
| Mazzone, 2013 | Low | <https://doi.org/10.1093/europace/eut126> | 48 | 81 | 65.4 | 11 (22.9) | 44/48 (91.7) | 47/47 (97.9) | 2 (4.2) | 0 (0.0) |
| Migliore, 2019 | High | <https://doi.org/10.1111/pace.13700> | 198 | 393 | 69 | 39 (19.7) | 192/198 (97.0) | 196/198 (99.0) | 1 (0.5) | 0 (0.0) |
| Oto, 2012 | High | [10.1111/j.1540-8159.2012.03385.x](https://doi.org/10.1111/j.1540-8159.2012.03385.x) | 66 | 140 | 55.6 | 22 (33.3) | 58/66 (87.9) | 65/66 (98.5) | 1 (1.5) | 0 (0.0) |
| Preda, 2025 | High | <https://doi.org/10.1111/pace.15146> | 202 | 471 | 70 | 82 (40.6) | 449/471 (95.3) | 196/202 (97.0) | 0 (0.0) | 0 (0.0) |
| Qin - mechanical, 2021 | Low | <https://doi.org/10.1111/pace.14206> | 22 | 45 | 66.9 | 8 (36.4) | 42/45 (93.3) | 45/45 (100.0) | 1 (4.5) | 0 (0.0) |
| Refaat, 2019 | Low | DOI: 10.12816/0053741 | 43 | 88 | 59 | 8 (18.6) | 38/43 (88.4) | 43/43 (100.0) | 0 (0.0) | 0 (0.0) |
| Sharma, 2018 | High | <https://doi.org/10.1016/j.jacep.2017.12.010> | 400 | 683 | 71.2 | 117 (29.3) | 399/400 (99.8) | 663/683 (97.1) | 6 (1.5) | 0 (0.0) |
| Starck, 2020 | High | <https://doi.org/10.1093/europace/euaa103> | 992 | 1552 | 66 | 298 (30.0) | 1478/1552 (95.2) | Na | 16 (1.6) | 4 (0.4) |
| Villegas, 2022 | High | <https://doi.org/10.1111/pace.14625> | 826 | 1227 | 67 | 285 (34.5) | 820/826 (99.3) | 824/826 (99.8) | 2 (0.2) | 0 (0.0) |
| Williams, 2025 | High | [10.1016/j.xjon.2024.11.010 External Link](https://doi.org/10.1016/j.xjon.2024.11.010) | 104 | 210 | 63.8 | 32 (30.8) | 99/104 (95.2) | na | 2 (1.9) | 2 (1.9) |
| Witte - Evolution 2017 | Low | [10.1093/europace/euw255](https://doi.org/10.1093/europace/euw255) | 50 | 56 | 68 | 15 (30.0) | 45/56 (80.4) | 55 | 0 (0.0) | 0 (0.0) |
| Witte - Evolution RL 2017 | Low | [10.1093/europace/euw255](https://doi.org/10.1093/europace/euw255) | 53 | 93 | 65 | 14 (26.4) | 90/93 (96.8) | 92 | 0 (0.0) | 0 (0.0) |
| Zsigmond - mechanical, 2023 | Low | <https://doi.org/10.1093/europace/euac200> | 49 | 86 | 62.6 | 8 (16.3) | 71/86 (82.6) | 42 | 1 (2.0) | 0 (0.0) |

**Supplemental Table 2** List of rotational TLE studies


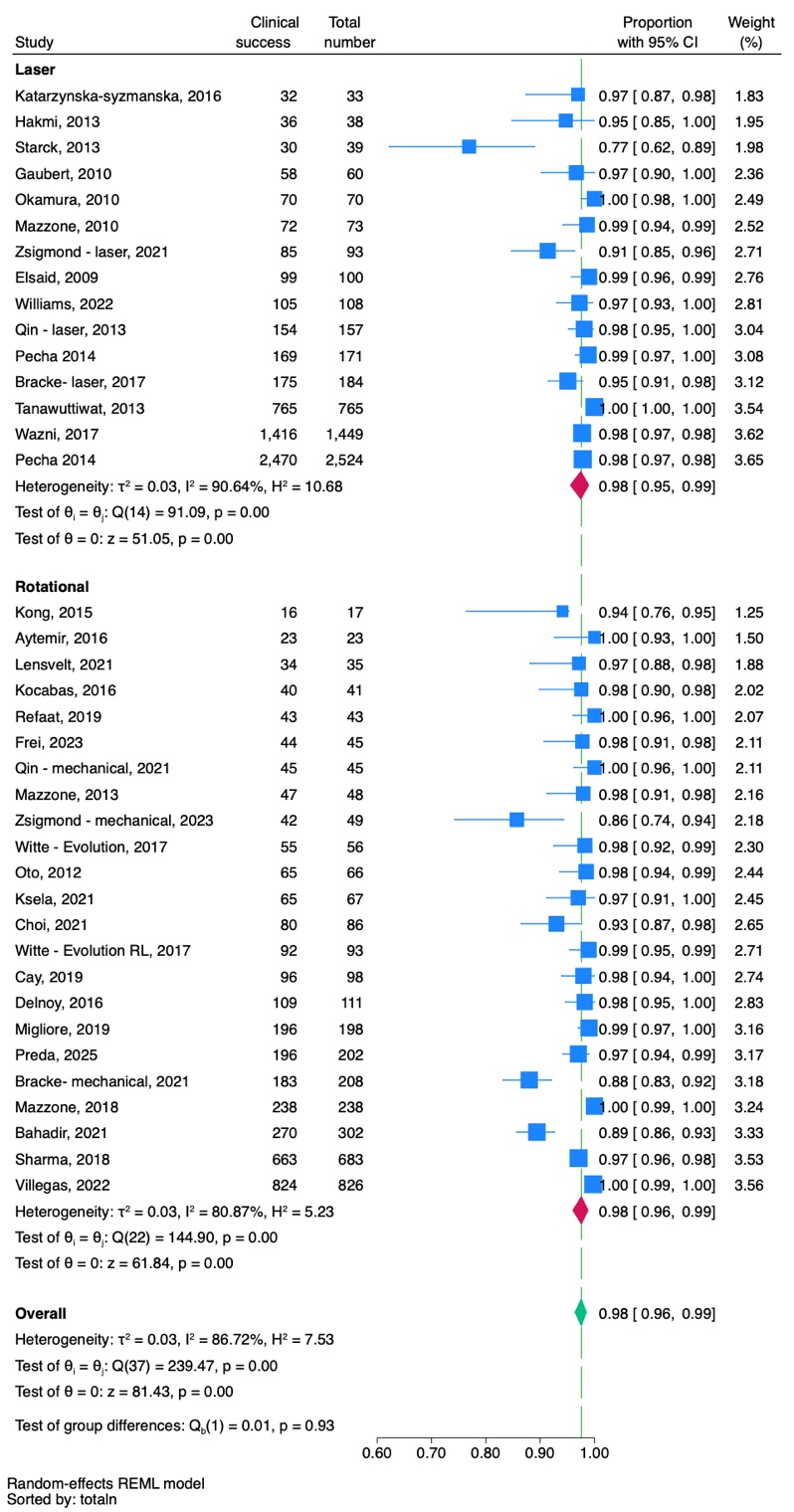


**Supplemental Figure 1** Forest plot comparing clinical success rates between laser and rotational TLE


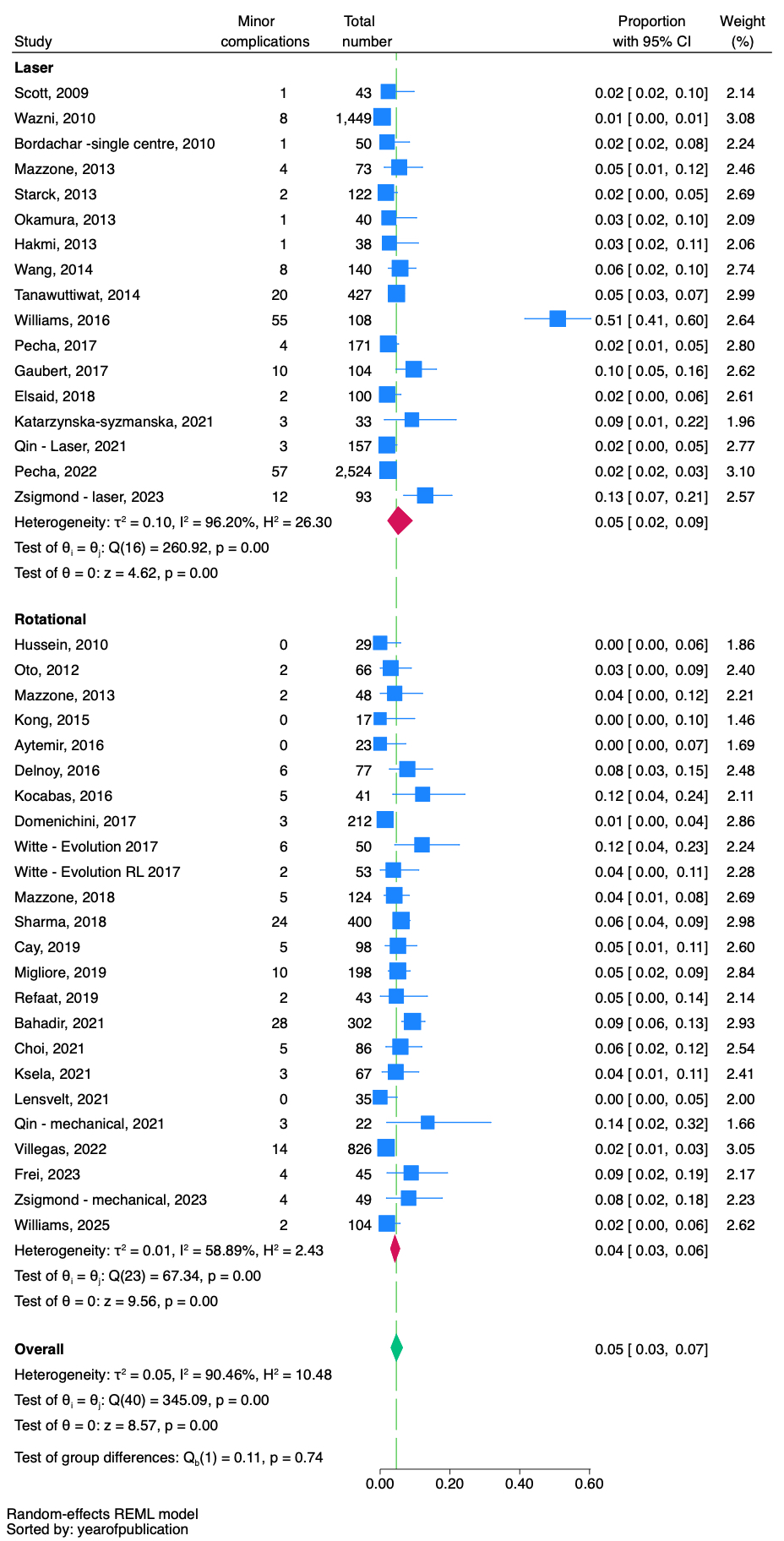


**Supplemental Figure 2** Forest plot comparing minor complication rates between laser and rotational TLE


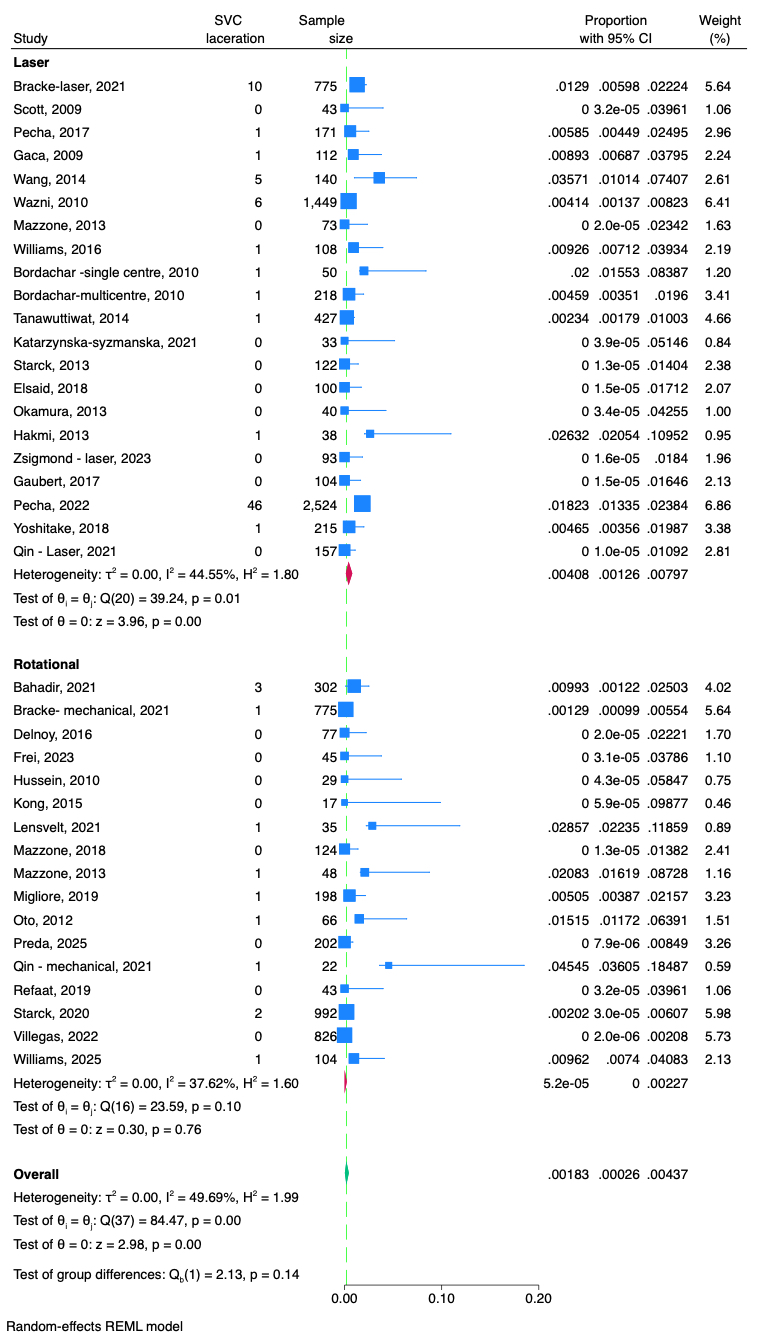


**Supplemental Figure 3** Forest plot comparing SVC laceration rates between laser and rotational TLE.


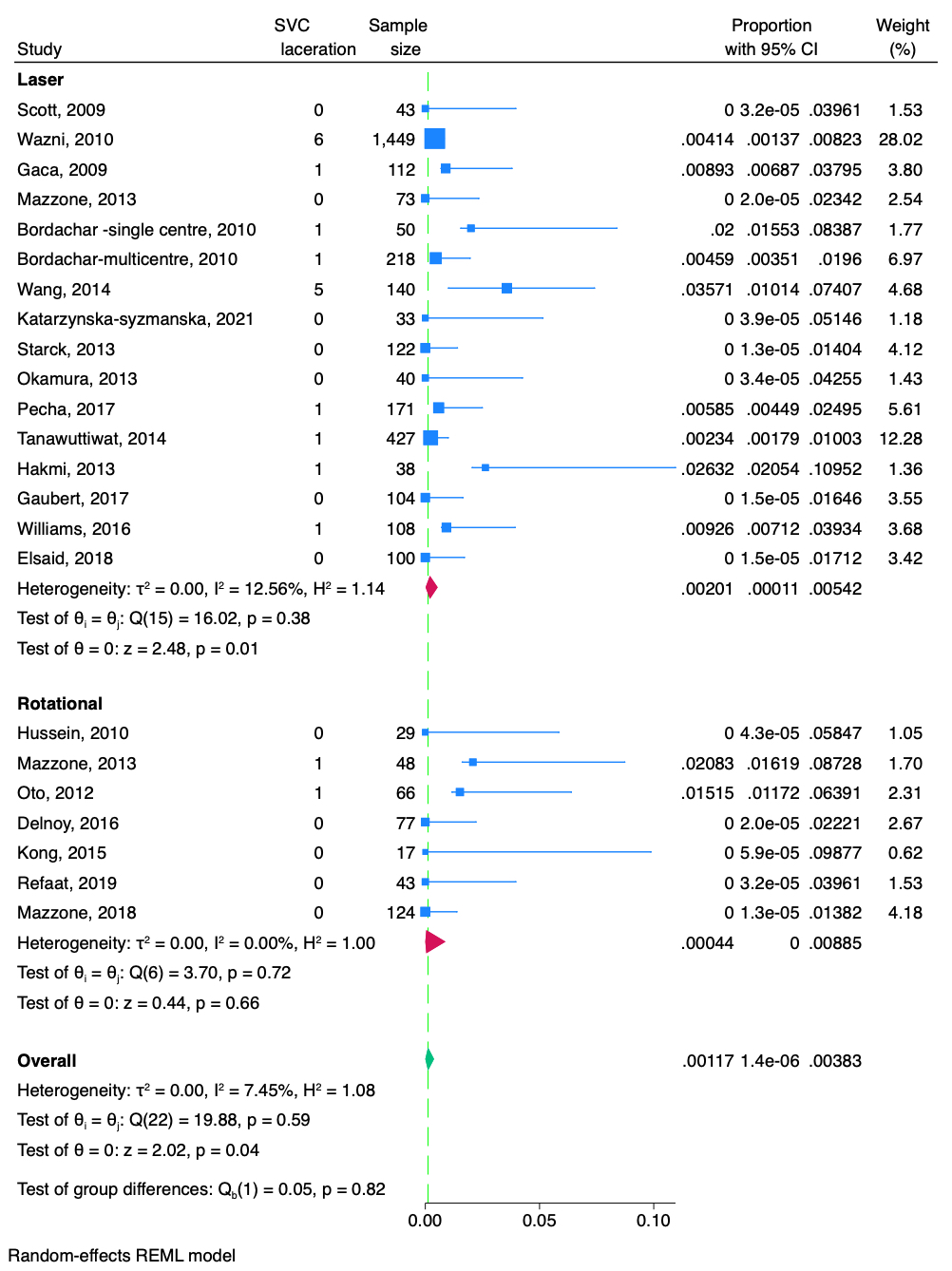


**Supplemental Figure 4** Forest plot comparing SVC laceration rates between laser and rotational TLE in studies which included patients up to 2016.


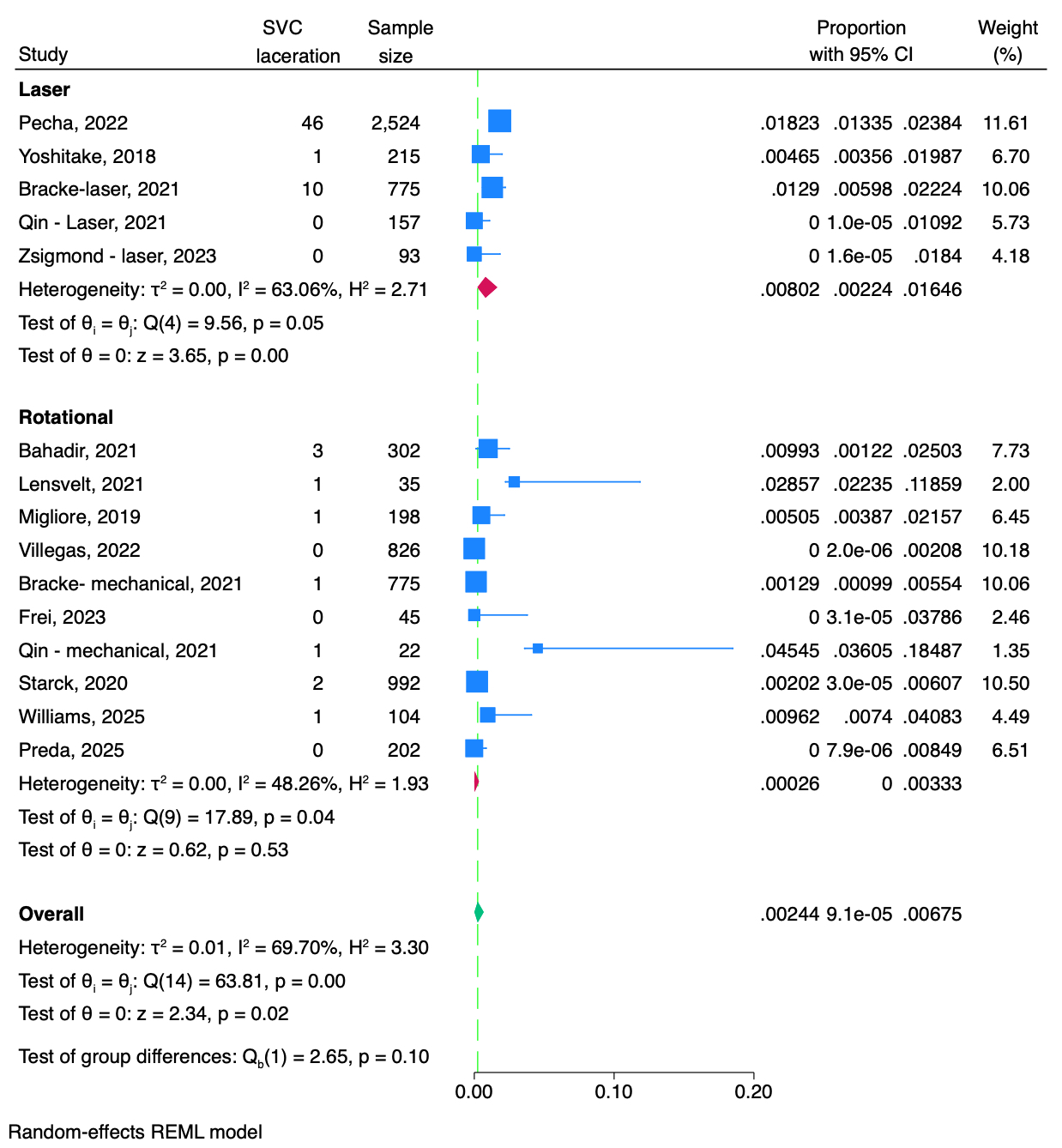


**Supplemental Figure 5** Forest plot comparing SVC laceration rates between laser and rotational TLE in studies which included patients after 2016.


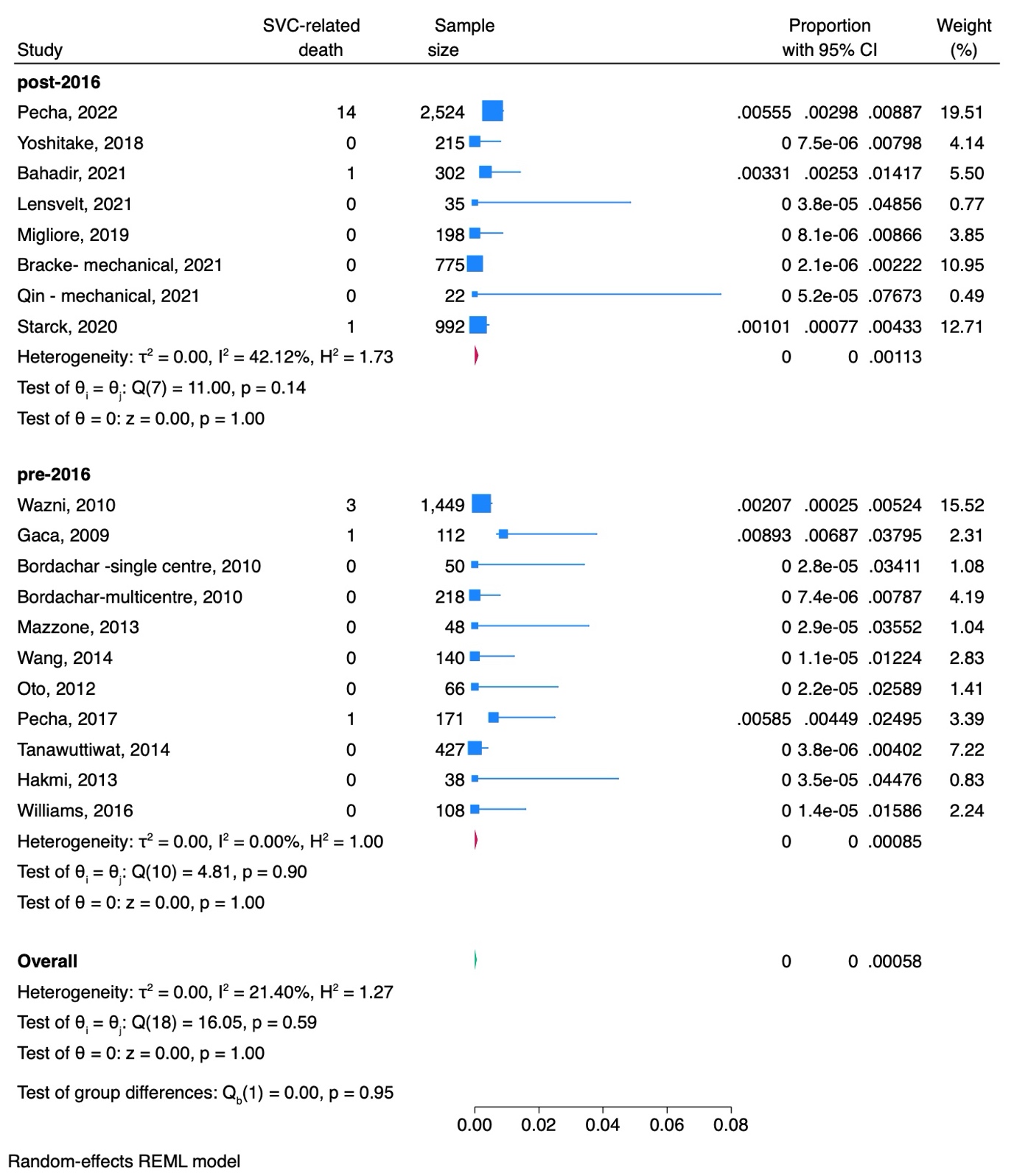


**Supplemental Figure 6** Forest plot of subgroup analysis comparing SVC-related deaths between pre-2016 and post-2016 studies.


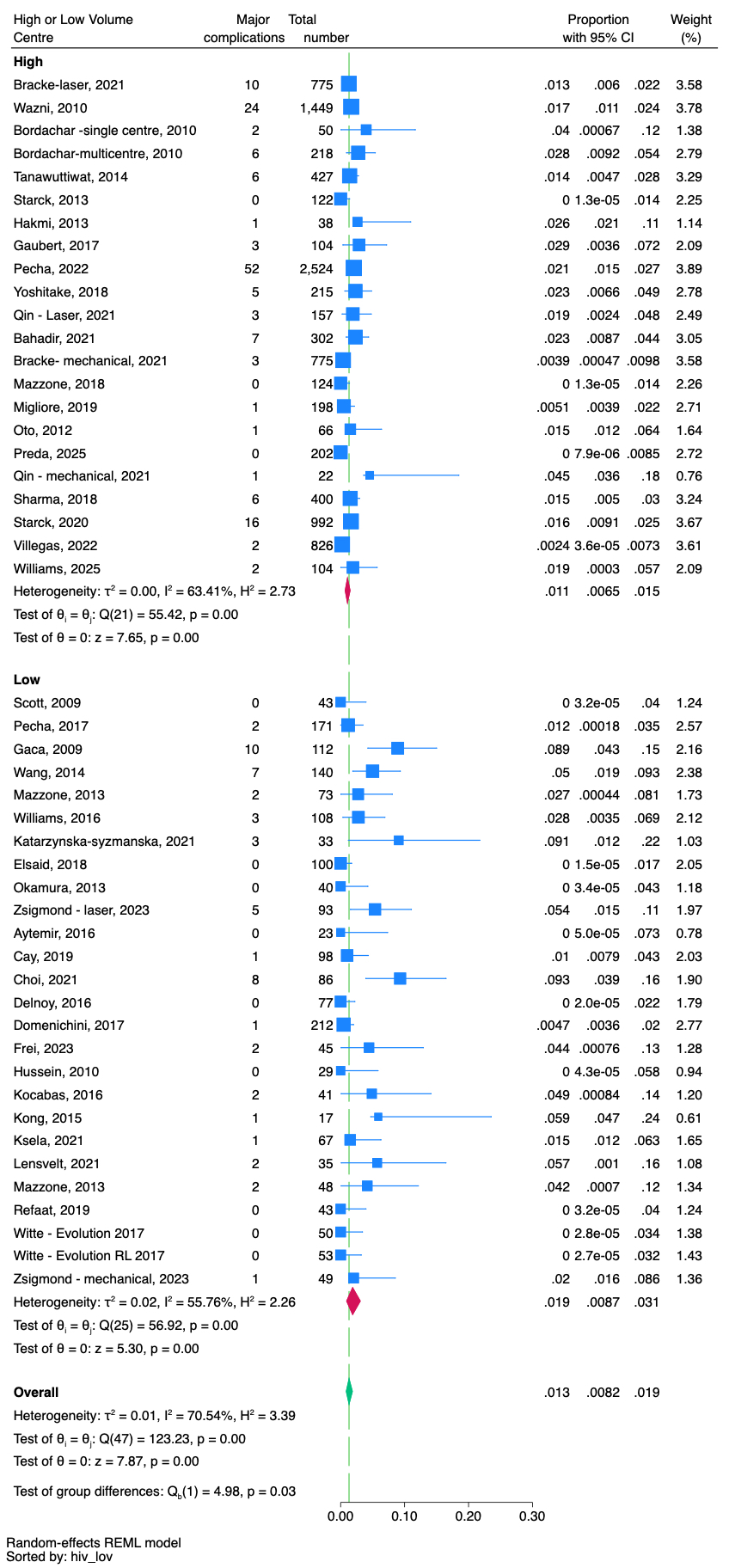


**Supplemental Figure 7** Forest plot of subgroup analysis comparing major complication rates between high-volume centers and low-volume centers.


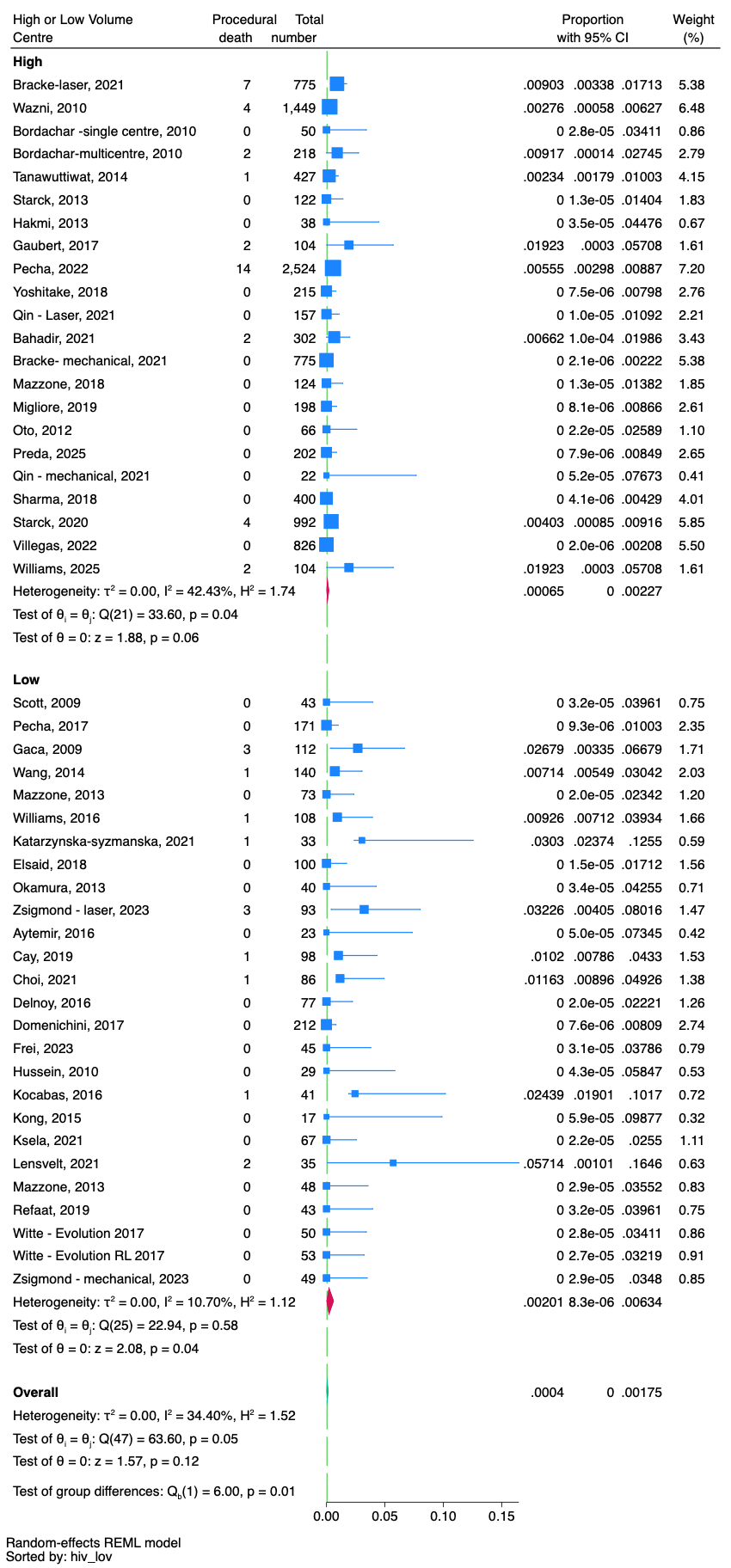


**Supplemental Figure 8** Forest plot of subgroup analysis of procedural deaths between high-volume centers and low-volume centers.
